# Momentum-Resolved Signatures of Carrier Screening Effects on Electron–Phonon Coupling in MoS_2_

**DOI:** 10.1021/acsnano.5c00744

**Published:** 2025-03-15

**Authors:** Yiming Pan, Patrick-Nigel Hildebrandt, Daniela Zahn, Marios Zacharias, Yoav William Windsor, Ralph Ernstorfer, Fabio Caruso, Hélène Seiler

**Affiliations:** † Institut für Theoretische Physik und Astrophysik, Christian-Albrechts-Universität zu Kiel, 24118 Kiel, Germany; ‡ Fritz Haber Institute of the Max Planck Society, Faradayweg 4-6, 14195 Berlin, Germany; § Univ Rennes, INSA Rennes, CNRS, Institut FOTON—UMR 6082, F-35000 Rennes, France; ∥ Institut für Optik und Atomare Physik, Technische Universität Berlin, Strasse des 17. Juni 135, 10623 Berlin, Germany; ⊥ Freie Universität Berlin, Arnimallee 14, 14195 Berlin, Germany

**Keywords:** carrier screening, electron−phonon coupling, ultrafast electron diffraction, first-principles calculations, layered materials

## Abstract

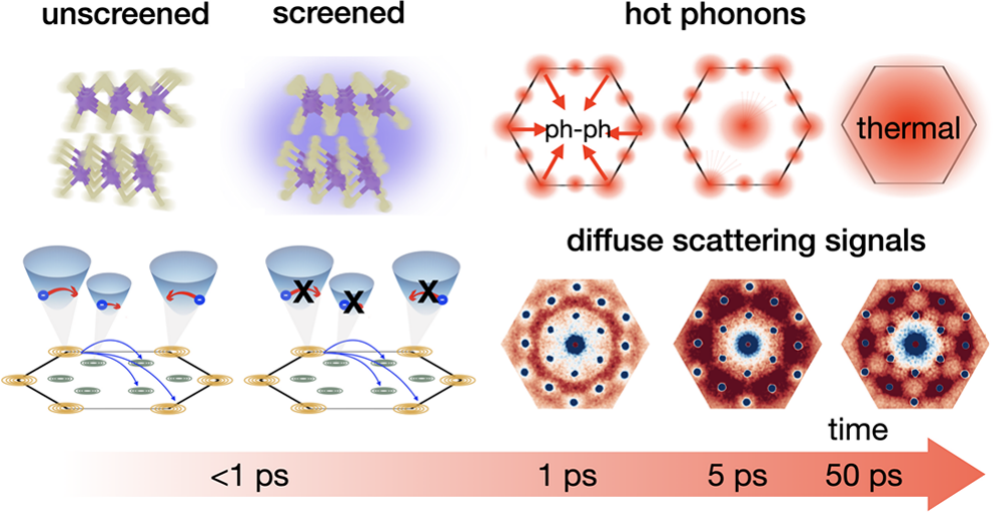

Electron–phonon coupling is central to many condensed matter phenomena. Harnessing these effects for functionality in materials always involves nonequilibrium electronic states, which in turn alter quasi-free-carrier density and screening. Thus, gaining a fundamental understanding of the interplay of carrier screening and electron–phonon coupling is essential for advancing ultrafast science. Prior work has mainly focused on the impact of carrier screening on electronic structure properties. Here, we investigate the nonequilibrium lattice dynamics of MoS_2_ after a photoinduced Mott transition. The experimental data are closely reproduced by ab initio ultrafast dynamics simulations. We find that the nonthermal diffuse scattering signals in the vicinity of the Bragg peaks, originating from long-wavelength phonon emission, can only be reproduced upon explicitly accounting for the screening of electron–phonon interaction introduced by the Mott transition. These results indicate that carrier screening influences electron–phonon coupling, leading to a suppression of intravalley phonon-assisted carrier relaxation. Overall, the combined experimental and computational approaches introduced here offer prospects for exploring the influence of screening of the electron–phonon interactions and relaxation pathways in driven solids.

Carrier screening has emerged as a powerful control knob of materials’ properties, both statically and on ultrafast time scales.^1^ At high excitation densities, the electronic excitations generated by a femtosecond laser pulse significantly modify the dielectric environment via carrier screening. A well-known consequence of such ultrafast photodoping is bandgap renormalization, an effect that has been investigated for decades as observed in many materials, ranging from semiconductors to quantum materials.^2−9^ Recently, it has been demonstrated that dynamical screening can also be the origin of emergent electronic phases that are only accessible out of equilibrium.^10−12^ For example, carrier screening was predicted to modify the effective electronic correlations dynamically,^13,14^ and leads to an ultrafast Lifschitz transition in MoTe_2_ as observed in photoemission experiments.^11^ In semiconducting transition metal dichalcogenides (TMDCs), carrier screening leads to the decrease of the attractive interactions of the strongly bound excitons and a reduction of the exciton binding energy.^15,16^ Beyond certain carrier densities, a Mott transition where the excitons are quenched and dissociate into free electron and hole plasma occurs.^2,5,17−22^ Overall, these examples demonstrate that femtosecond laser pulses offer a way to alter a material’s response via dynamical screening on ultrafast time scales.

Prior work has mostly focused on the impact of carrier screening on the properties of electronic structures. Much less is known regarding the effects of carrier screening on the lattice interactions. Recently, dielectric screening from the substrate has been shown to significantly alter the phonon dynamics in monolayer MoS_2_.^23^ However, to the best of our knowledge, experimental evidence of how carrier screening within a material modifies electron–phonon coupling has remained scarce. From the theory side, the renormalization of electron–phonon coupling (EPC) matrix elements due to screening effects suggests that the lattice dynamics can be affected by changes in the dielectric environment.^23−26^ Furthermore, the screening introduced by the electron–hole plasma is particularly effective for lattice vibrations which create macroscopic electric fields of wavelength longer than the screening length,^27−29^ modifying the interactions between electron and lattice and possibly allowing access to hidden quantum phases of the lattice.^30,31^ Understanding how electron–phonon coupling and the lattice dynamics are impacted by carrier screening is therefore relevant both at the fundamental level and in view of devising new lattice control schemes.

Here we induce a Mott transition in bulk MoS_2_ with an ultrashort laser pulse and directly investigate the lattice dynamics in momentum space using a combination of femtosecond electron diffuse scattering (FEDS) and first-principles calculations, which include free-carrier screening to the EPC matrix elements. Working with bulk MoS_2_ enables us to avoid screening effects from the substrate^23^ and to focus on carrier screening that is intrinsic to the material. Surprisingly, our data reveal that lattice relaxation involves two qualitatively distinct nonthermal phonon populations. Ultrafast dynamics simulations using unscreened EPC matrix elements successfully capture short-wavelength phonon emission and lattice relaxation for time delays beyond 5 ps. On shorter time scales, however, the diffuse scattering signals in the vicinity of the Bragg peaks can only be reproduced upon explicitly accounting for the screening of the electron–phonon interactions in ab initio calculations. These findings indicate that screening affects primarily long-wavelength phonons around the Γ point and leads to a suppression of intravalley scattering pathways. Overall, our results suggest that carrier screening profoundly alters materials’ electron–phonon coupling and lattice relaxation pathways, providing access to hidden dynamical regimes. Given that electron–phonon coupling and screening are ubiquitous in condensed matter, our findings are generally applicable. Exploring such EPC renormalization in the future offers new opportunities for devising schemes to control lattice properties on ultrafast time scales, which ultimately could lead to new functionalities in quantum technologies and optoelectronic devices.

## Results

**Figure 1:**
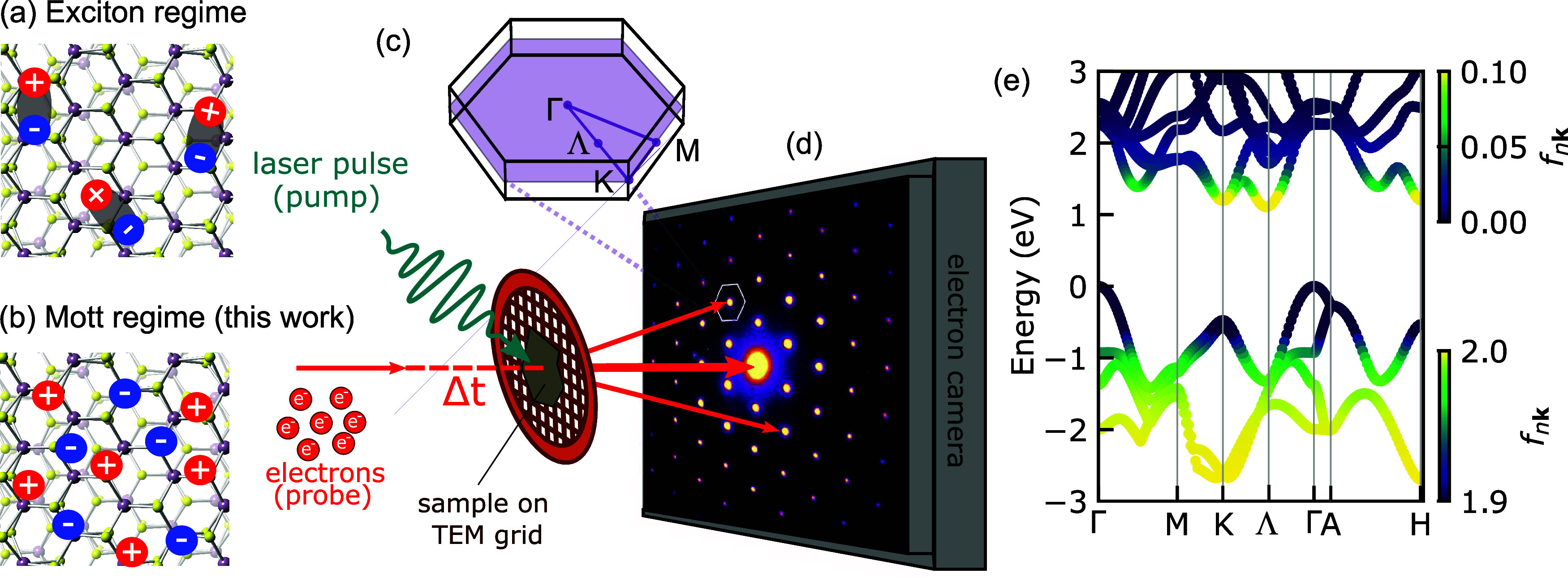
Crystal structure of multilayer MoS_2_ and schematic illustration of (a) exciton regime at low carrier excitation densities and (b) Mott regime at high carrier excitation densities (>1.5 × 10^13^ cm^–2^). (c) Brillouin zone (BZ) and high-symmetry points of bulk MoS_2_. (d) Schematic illustration of the FED experiment. (e) Calculated electron band structure of bulk MoS_2_ and initial distribution of photoexcited carriers superposed as a color coding.

The layered hexagonal structure of bulk MoS_2_ is shown from the top in Figure 1(a,b), whereas the three-dimensional (3D) Brillouin zone (BZ) is displayed in Figure 1(c) along with high-symmetry points. In our diffraction experiment, we obtained a direct view of the reciprocal lattice for momenta within the Γ-K-M plane in the BZ (shaded purple plane in Figure 1(c)). Each Bragg peak acts like the center of a first BZ, located at the Γ point. An exemplary diffraction pattern obtained on a mechanically exfoliated MoS_2_ flake of thickness ≃35 nm is shown in Figure 1(d), which illustrates the basic idea of an FED experiment.^32^ Here we employ ≈ 50 fs, 2.1 eV pump pulses to excite the MoS_2_ flake. We estimate our excitation density to be in the range of 1.29–1.69 · 10^14^ cm^–2^, well into the Mott regime^2,4,5,19,20^ (see Section 1 in the Supporting Information (SI)^33^). Therefore, optical excitation quickly leads to the formation of an electron–hole plasma, and exciton effects are suppressed by screening, as depicted in Figure 1(a,b). Following the generation of electrons and holes, electron–phonon coupling transfers energy from the photoexcited carriers to the crystal lattice. Because electron–phonon coupling varies for different modes, energy can be preferentially deposited in strongly coupled modes, leading to selected phonons with larger occupation number. In such case, the lattice cannot be described by Bose–Einstein statistics, resulting in a nonthermal phonon distribution. FEDS is ideally suited to probe nonthermal phonons due to its momentum information. Following the optical pump, the nonequilibrium lattice dynamics in the sample is probed by a 150 fs bunch of electrons which diffracts off the lattice. The FEDS signals vary linearly with phonon populations, and their transient changes directly reflect the time evolution of the nonthermal phonon populations at different wavevectors in the BZ. For instance, long-wavelength phonons affect the FEDS signals in the vicinity of the Bragg peaks close to Γ, while short-wavelength phonons induce changes at K or M. More details about FEDS can be found in refs (32,34−37). All measurements are carried out at room temperature.

Before discussing the lattice dynamics, we consider the initial populations of electrons and holes in more detail. Figure 1(e) displays the electronic band structure of bulk MoS_2_ calculated with density functional theory (DFT).^38^ In contrast to monolayer MoS_2_, bulk MoS_2_ is a semiconductor with an indirect bandgap of 1.29 eV between Γ and its conduction band minimum (CBM) located at Λ, around the midpoint between Γ and K.^39,40^ Within several tens of femtoseconds, the excited carriers thermalize within conduction bands and valence bands via electron–electron scattering.^41−43^ The superimposed color code on the band structure shows the initial (*t* = 0) thermalized distribution of photoexcited carriers *f*_*n***k**_ in the conduction and valence bands. These are simulated with Fermi–Dirac distributions with a hot carrier temperature (4000 K) and different chemical potentials for electrons and holes to fix the carrier density to the experimental conditions. The electronic temperature is chosen such that the final lattice temperature in the simulations match the estimated temperature rise in the experiments of Δ*T* = 70 K. The initial distribution of electrons and holes shows that in conduction bands primarily the K and Λ pockets are occupied by electrons and in valence bands the K and Γ are populated by holes after photoexcitation.^44^ Initial distribution with other carrier temperatures led to qualitatively similar results, with different final lattice temperatures. We discuss these results in Sections 2 and 3 of the SI.

**Figure 2:**
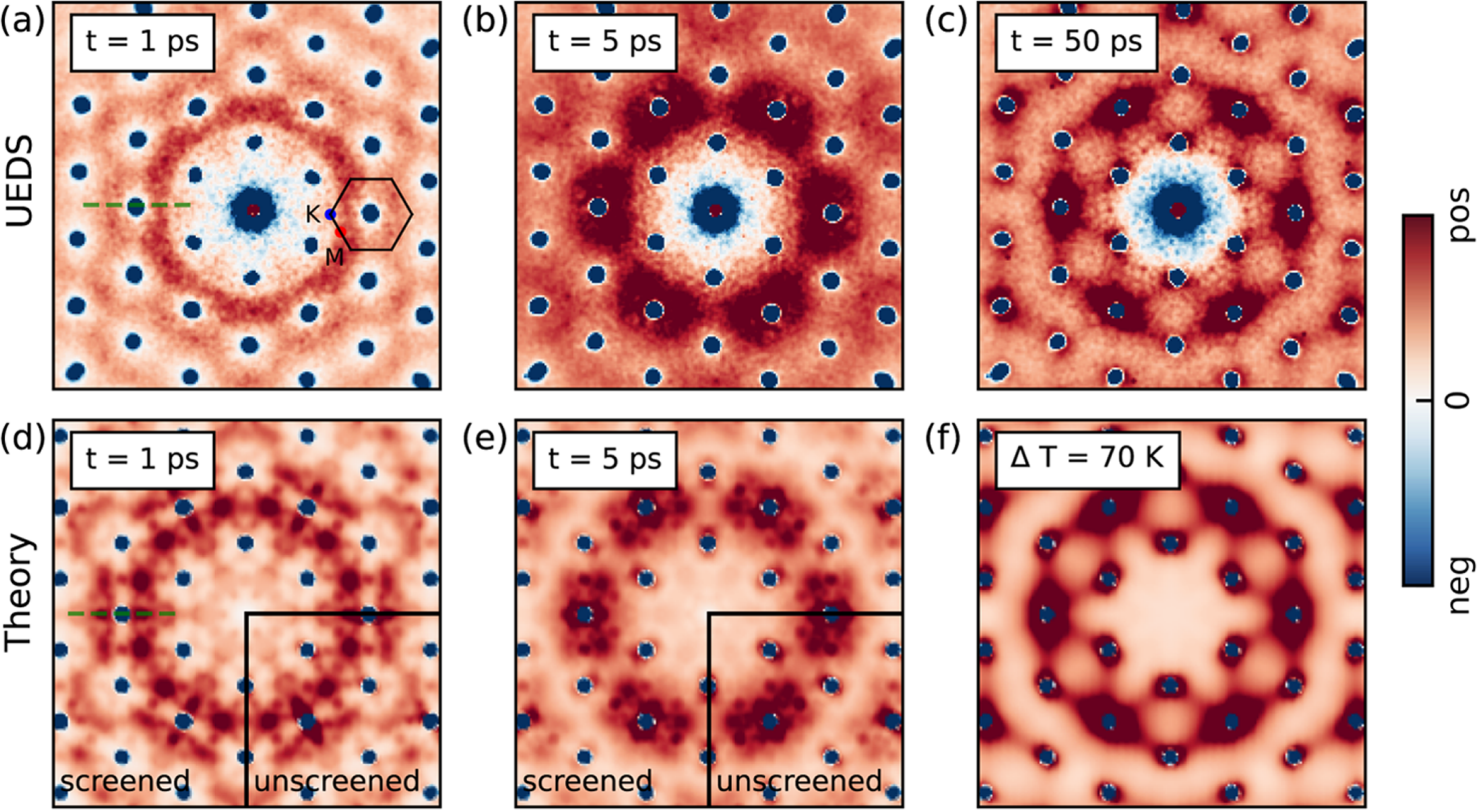
Differential diffuse scattering signals Δ*I*(**Q**, *t*) = *I*(**Q**, *t*) – *I*(**Q**, *t* ≤ *t*_0_) at pump–probe delays of (a) 1 ps, (b) 5 ps, and (c) 50 ps from FEDS experiments. Simulated diffuse scattering maps at pump–probe delays of (d) 1 ps with screened EPC matrix elements and unscreened EPC in the fourth quadrant of the map and (e) 5 ps with screened EPC matrix elements and unscreened EPC in the fourth quadrant of the map. (f) Thermal diffuse scattering pattern, Δ*I*(**Q**) = *I*(**Q**, *T* = 370 K) – *I*(**Q**, *T* = 300 K), where 370 K is the estimated final temperature and 300 K is the initial lattice temperature.

A detailed view of phonon dynamics across the BZ is obtained by inspecting inelastic scattering signals between the Bragg peaks via FEDS.^23,26,34−36,45,46^ Details are discussed in Section 4 of the SI. The Bragg peak dynamics are discussed in Section 5 of the SI.^47,48^ An overview of the FEDS signals from the experiments is shown in Figure 2. Here, the differential diffuse scattering signals Δ*I*(**Q**, *t*) are obtained by subtracting the diffraction intensity at pump–probe time delay *t* from the equilibrium diffraction pattern *I*(**Q**, *t* ≤ *t*_0_) before excitation. In panels (a-c), we present Δ*I*(**Q**, *t*) for pump–probe time delays of 1, 5, and 50 ps. These data demonstrate qualitative changes in the distribution of the inelastic signals as pump–probe delay increases, reflecting different phonon populations at different times. Strikingly, these data display signatures of two distinct nonthermal phonon distributions. The inelastic scattering distribution at 1 ps qualitatively differs from the one at 5 ps, which in turn differs from the distribution at 50 ps.

To gain further microscopic insights into the coupled electron–lattice dynamics seen in the experiments, we perform ab initio calculations of the nonequilibrium diffuse scattering. We simulate the real-time dynamics of the coupled electron and phonon systems by solving the time-dependent Boltzmann Equation (TDBE).^49−56^ We use the initial carrier distribution as discussed in Figure 1(e) and initial phonon population with a thermal distribution at 300 K. These results are then used as inputs for computations of the all-phonon structure factor in nonequilibrium.^37,57,58^ This approach enables direct comparison of the theoretical and experimental data.^23,26,36^ The simulation results are shown in Figure 2(d–f), and a movie with a comparison between simulations and experiments at all pump–probe delays is available in the Supporting Information.

We perform simulations with and without carrier screening effects arising from the Mott transition induced by the intense driving field in the FEDS experiments. The influence of photoexcited carriers on phonon dynamics is included by screening the EPC matrix elements^24,25,27,28,59−61^*via g̃*_*mnν*_(**k**, **q**) = ϵ_MT_^–1^(**q**)*g*_*mnν*_(**k**, **q**), where *g*_*mnν*_(**k**, **q**) are the EPC matrix elements without carrier doping obtained from density functional perturbation theory.^62^ ϵ_MT_^–1^(**q**) accounts for additional screening components to phonons of wave vector **q**, arising from the Mott transition and the photoexcited free carriers. In the independent-particle approximation:
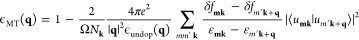
1where δ*f*_*m***k**_ accounts for the change of carrier occupations due to optical excitation. The electron energies ε_*m***k**_ and the periodic part of the Bloch eigenvectors |*u*_*m***k**_⟩ are obtained from DFT and Wannier interpolation techniques.^63,64^ ϵ_undop_ indicates the dielectric function of undoped MoS_2_. Equation 1 and its numerical evaluation are discussed in Section 6 of the SI.

**Figure 3:**
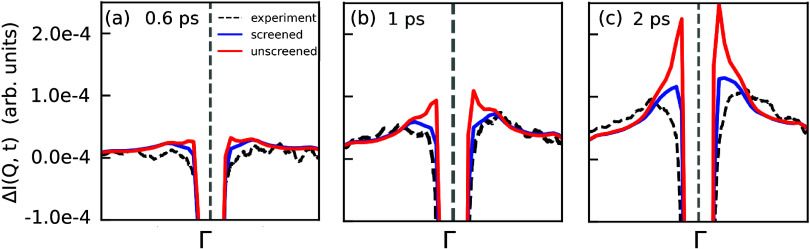
Transient diffuse scattering intensity at (a) 0.6, (b) 1.0, and (c) 2.0 ps in the vicinity of the (110) Bragg peak for unscreened (red) and screened (blue) simulations and experiments (dashed black). The phonon momentum on the *x* axis follows the path marked by the dashed green lines in Figure 2(a,d).

The comparison of the experimental and simulated data in Figure 2 shows that the ab initio simulations successfully capture the two-stage lattice relaxation observed in the experiments. Finer structure features are seen in the theory maps, which we cannot capture in our experiments due to signal-to-noise limitations. The influence of carrier screening on the diffuse scattering signals can be determined by comparing the diffuse scattering maps computed with the nonequilibrium phonon populations obtained with screened and unscreened EPC matrix elements. In Figure 2(d), the fourth quadrant is the result with unscreened EPC and the other three quadrants are the results with screened EPC. Overall, the differences between the screened and unscreened cases are mainly visible around the Bragg peak area, while higher momenta of the BZ remain largely unaffected. Furthermore, we observe that the differences between unscreened and screened cases are largely reduced by 5 ps; see Figure 2(e). To qualitatively assess the influence of screening on the emission of long-wavelength phonons, we report in Figure 3 the FEDS intensity along a path in reciprocal space at different time delays, marked by the green dashed line in Figure 2(a,d). In the absence of carrier screening, simulations predict a sharp rise of the FEDS intensity close to the Bragg peaks due to the large phonon populations emitted through intravalley relaxation pathways. Upon accounting for screening of the electron–phonon coupling, conversely, the emission of long-wavelength phonons is strongly suppressed, leading to a substantial reduction of the FEDS intensity in the vicinity of the Bragg peaks. Overall, the inclusion of screening is crucial to qualitatively reproduce the experimental trend. These results open new prospects for exploring the influence of screening on the lattice dynamics through the combination of FEDS experiments and ab initio calculations in the vicinity of the Bragg peaks.

**Figure 4:**
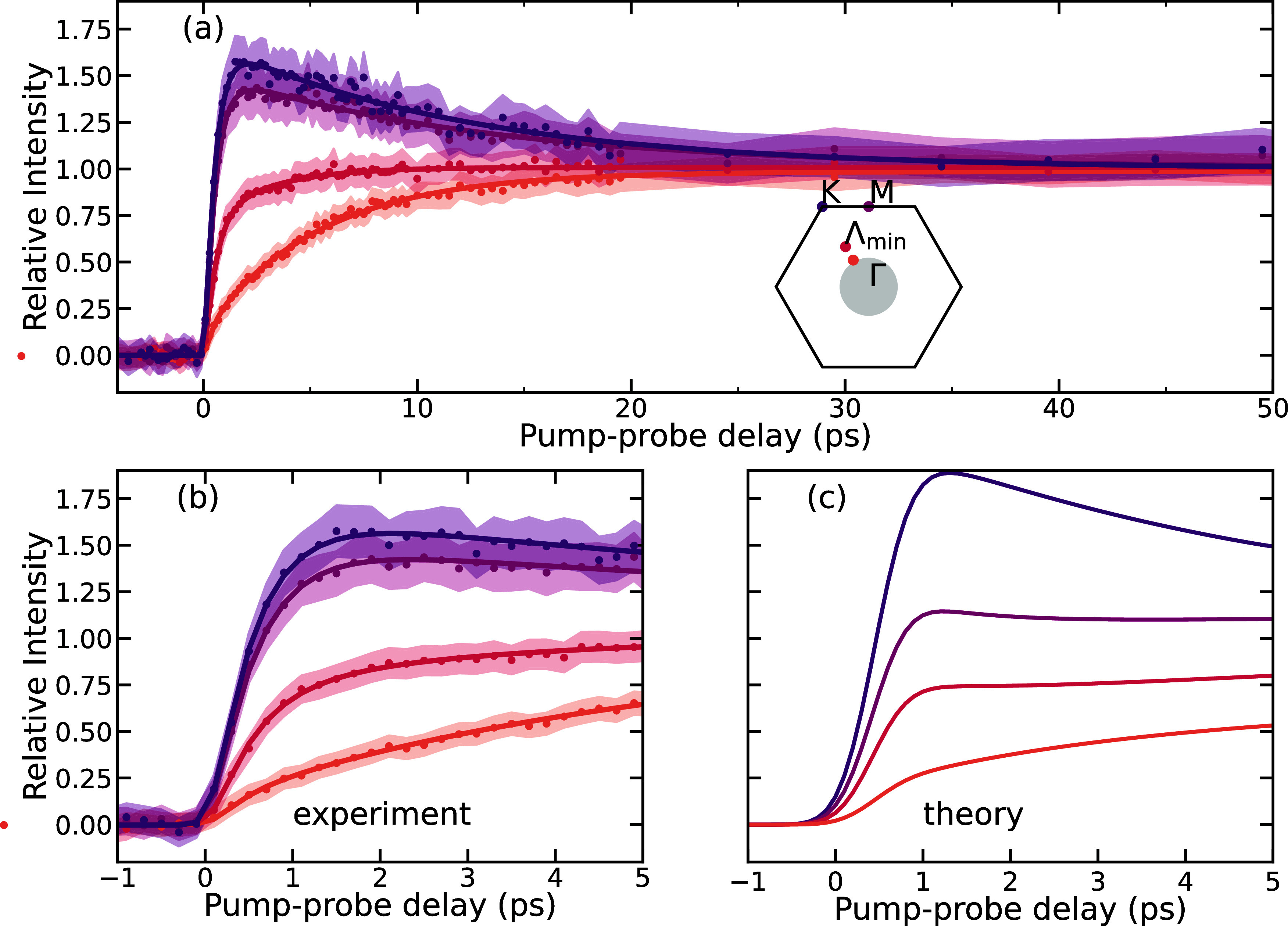
(a) Time-resolved relative differential diffuse scattering intensity at selected **q** points along the Γ-Λ-K high-symmetry path as well as for the M point, indicated as colored dots on the BZ in the inset. The shaded areas correspond to the standard error of the mean signal over multiple delay scans. The relative intensities are normalized to the late time signals at 50 ps and are fitted with a biexponential function (continuous lines). (b) Same as panel (a) for the first 5 ps. (c) Simulated relative differential diffuse scattering intensities for the same **q** points as in (a, b), matching colors. The simulated traces were convolved with a Gaussian function corresponding to the finite IRF of the experiments.

Quantitative information on the nonequilibrium phonon dynamics can be retrieved via a momentum-resolved analysis of the diffuse scattering signals. It is desirable to extract the relative intensity at specific momenta. Figure 4 displays relative diffuse scattering intensity at selected q points in the BZ (see Section 7 of the SI for more details about the extraction of the signals). The fastest and strongest amplitude rise are observed for M and K phonons, with time constants of 0.43 ± 0.02 ps and 0.55 ± 0.02, respectively, matching well with the fast time constant measured in the Bragg peak dynamics. From our simulations, we retrieve consistent relative intensity amplitudes. We find that the M point dynamics is 0.12 ps, while the K point dynamics is 0.18 ps. In the experiments, the instrument response function (IRF) can only be estimated and not directly measured, which may cause some variations in the extracted time constants and may explain the faster time constants observed in the simulations. The rise is followed by a similar decay with a time constant of 11.8 ± 0.8 and 11.6 ± 0.7 ps for M and K phonons, respectively. Following the fast rise of K and M phonons, the phonon dynamics at Λ can be fitted with two rising exponentials, with time constants of 0.6 ± 0.1 and 3.1 ± 0.5 ps. We observe that the closer we approach Γ, the slower the phonon population rises. The experimental traces shown in Figure 4(b) can be directly compared with the temporal evolution of the relative intensities from our ab initio computations, shown in panel (c). We make a quantitative analysis of the nonequilibrium lattice dynamics by tracking the relative intensity as a function of delay time for 3 points along the Γ–K high-symmetry path (see Section 7 of the SI) as well as at the M point. The comparison of simulated and experimental relative intensities shown in Figure 4(b,c) demonstrates an agreement between the FEDS experiments and TDBE simulations.

**Figure 5:**
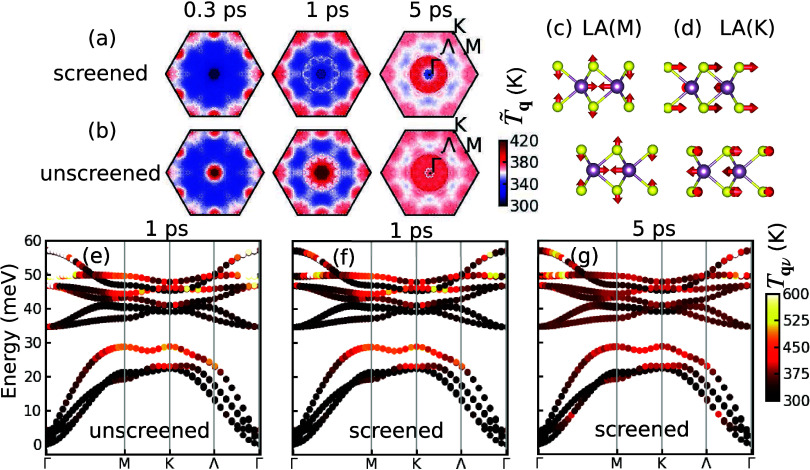
Effective phonon temperature *T̃*_**q**_ at 0.3, 1, and 5 ps simulated with screened (a) and unscreened (b) EPC matrix elements. (c) Eigenmode of LA(M) and (d) LA(K) phonons. (e) Branch- and momentum-resolved phonon temperature *T*_**q**ν_ superposed on the phonon dispersion along the high-symmetry path Γ-M-K-Γ at 1 ps calculated with unscreened EPC matrix elements. The same but with screened EPC matrix elements is shown for 1 ps (f) and 5 ps (g).

Next we show that carrier screening profoundly influences the nonthermal relaxation pathways by suppressing specific phonon-assisted recombination channels. These mechanisms modify the two-stage nonthermal phonon populations observed in our FEDS experiments. Figure 5 summarizes the nonequilibrium phonon dynamics obtained from our simulations. As an indicator of the nonthermal phonon populations established out of equilibrium, we introduce the effective phonon temperature, *T̃*_**q**_ = *N*_*ph*_^–1^ ∑_ν_*T*_**q**ν_, where *T*_**q**ν_ = ℏω_**q**ν_{*k*_B_ ln[1 + *n*_**q**ν_^–1^]}^−1^, and *n*_**q**ν_ and ω_**q**ν_ are the phonon distribution and frequencies, respectively. Figure 5(a,b) reports values of *T̃*_**q**_ for momenta in the BZ at time delays of *t* = 0.3, 1, and 5 ps. The results in panel (a) have been obtained by including carrier screening of the EPC induced by the photoinduced Mott transition via eq 1, whereas these effects are omitted in panel (b). These two regimes reveal large qualitative differences in the nonequilibrium dynamics of long-wavelength phonons, in particular <5 ps. In the unscreened case, the population of phonons close to Γ increases rapidly within 1 ps, revealing carrier relaxation pathways mediated by intravalley electron scattering. Conversely, in the presence of screening, intravalley scattering is inhibited, resulting in suppression of phonon emission around Γ. Long-wavelength phonons are only subsequently populated via phonon–phonon scattering on a time scale of 5 ps. The differences between screened and unscreened cases are further manifested by the mode- and momentum-resolved effective phonon temperatures *T*_**q**ν_, superimposed to the phonon dispersion curve in Figure 5(e,f) for *t* = 1 ps. These results reveal that screening affects primarily the dynamics of long-wavelength phonons, which is critical to retrieve the diffuse scattering fingerprints close to the Bragg peaks, as demonstrated by comparing the one-dimensional (1D) cuts shown in Figure 3(a–c) for the unscreened, screened, and experimental cases. The screening affects less the emission of LA phonons at M and K points, which contributes to the diffuse scattering due to the in-plane atomic motions for these two modes, as demonstrated by their eigenvectors in Figure 5(c,d).

**Figure 6:**
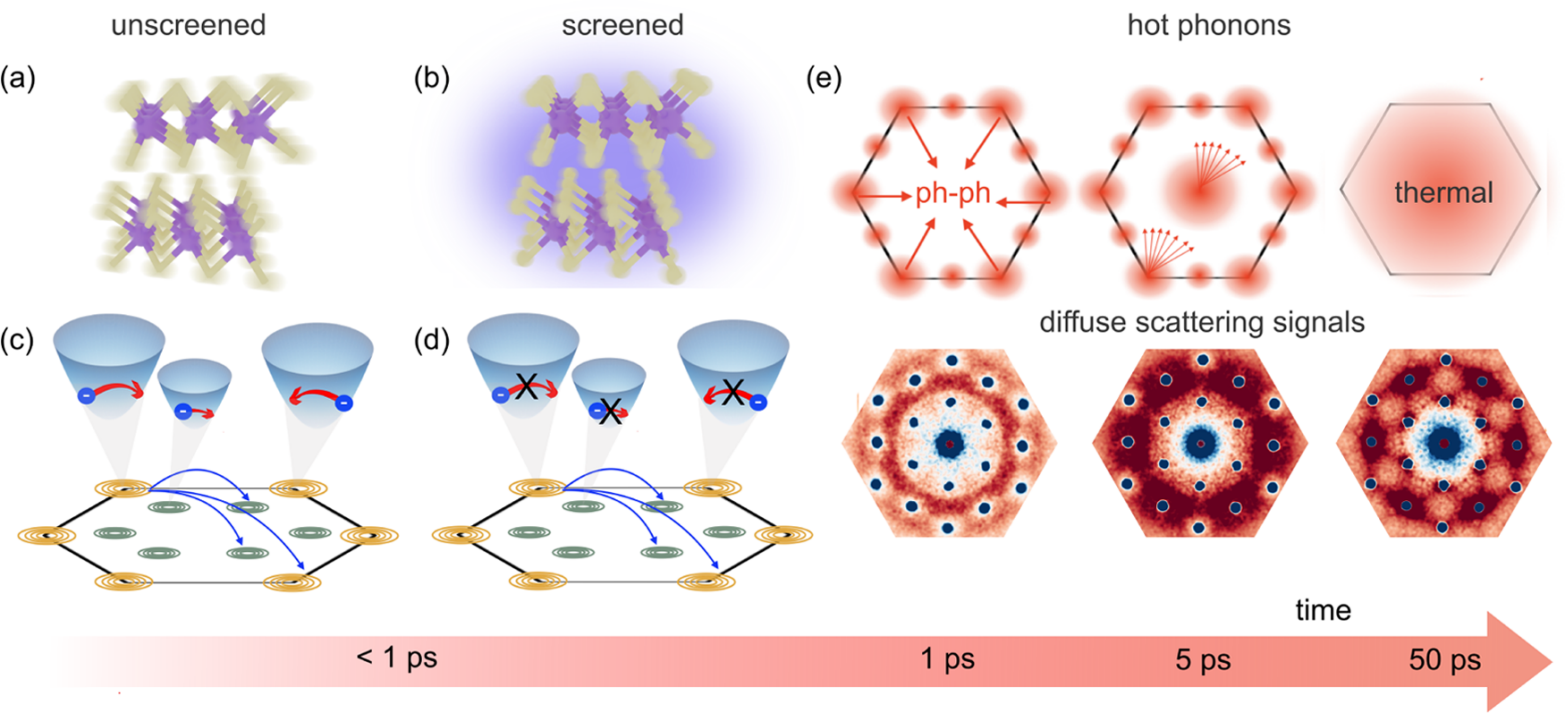
Impact of carrier screening on electron–phonon coupling and resulting modifications of the nonthermal lattice relaxation pathways. (a, b) Phonon emission close to the BZ center is strongly reduced as a result of carrier screening. This is illustrated for an exemplary high-frequency, long-wavelength optical phonon mode, which shows decreased fluctuations of atomic motions in the screened case. (c, d) Screening suppresses intravalley scattering of carriers, while intervalley scattering pathways are preserved. (e) Phonons are first excited at the BZ edge on the 1 ps time scale. These phonons are then scattered to the center of BZ on the 5 ps time scale. Finally, a quasi-thermalized state of the lattice is reached at 50 ps. The diffuse scattering signals corresponding to the hot phonon populations are shown below.

Bringing the results of Figures 2–6 together, we obtain a microscopic picture of how the momentum-dependent renormalization of the EPC due to screening changes the course of nonthermal phonon relaxation pathways in MoS_2_. We summarize the thermalization steps in Figure 6. We photoinduce a Mott transition in MoS_2_ using an ultrashort laser pulse. Electron–phonon intravalley scattering with low-momentum phonons (in the Γ–1/3K range) is strongly suppressed due to carrier screening, see the sketches in Figure 6(a,b). As a result, the first stage of the electron–phonon dynamics is dominated by intervalley scattering processes, which are unmodified by screening; see Figure 6(c,d). Inspecting the multivalley electronic band structure in Figure 1(e), one can expect K–K intervalley scattering in the conduction band and Γ-K scattering in the valence band to give rise to phonon emission at the K points of the BZ. Furthermore, K-Λ scattering processes in the conduction band are expected to produce significant phonon emission at the M points of the BZ, whereas the scattering between Λ points should give rise to phonon emission at K, Λ, and M. This is fully consistent with the experimental observations seen in Figure 4.

In the second stage of the phonon dynamics, phonon–phonon interactions lead to a more isotropic momentum-resolved temperature at 5 ps, as demonstrated in Figure 5(a,g). Nevertheless, the lattice remains in a nonthermal state, with minor differences persisting between the screened and unscreened cases. In this second stage, we attribute the origin of the nonthermal state of the lattice to two processes: (i) slower electron–phonon scattering of low-momentum phonons due to strong screening of the EPC for such phonons and (ii) slower phonon–phonon scattering rates of the acoustic branches in comparison with that of optical phonons, as illustrated by the anharmonic phonon scattering rate in Section 8 of the SI. A visible increase in phonon population around the Γ point is seen, but the TA and LA branches remain strongly out-of-equilibrium in momentum space. The thermalization limited by acoustic phonons seems to be a common feature in layered 2D materials, as investigated in recent works.^36,55,56,65^ Finally, at around 50 ps, the lattice reaches thermalization at an elevated temperature, as seen in Figure 2(c,f). These steps are illustrated schematically in Figure 6(e).

## Conclusions

Our study introduces a momentum-resolved approach to investigate the effect of carrier screening on electron–phonon coupling and provides first experimental evidence that large modifications of electron–phonon coupling are induced via photoinduced screening. Future advancements in FEDS will open new avenues for exploring screening dynamics. Enhanced beam coherence and improved signal-to-noise ratio in experiments will be particularly valuable in accessing regions closer to the Γ point, where the contrast between the screened and unscreened scenarios is expected to be most pronounced. These developments will further advance our understanding of screening, electron–phonon interactions, and their interplay in ultrafast phenomena. Electron–phonon coupling and carrier screening are both ubiquitous in condensed matter systems; thus, our findings can be generalized beyond MoS_2_. We expect carrier screening to similarly renormalize electron–phonon coupling for the long-wavelength phonons in other photoexcited materials at high excitation densities. Consequently, ultrafast photodoping can be used as a versatile tool to alter the nonthermal lattice relaxation pathways in materials, and our approach can be applied to diverse material platforms, ranging from semiconductors to quantum materials. We envision that control of electron–phonon interactions via carrier screening will give rise to advanced control schemes of materials’ properties, to reach hidden quantum phases that are not accessible in equilibrium or tuning properties, such as thermal or electrical conductivity on the ultrafast time scale.

## Methods

### Computational Details

The ground-state properties of bulk MoS_2_ are obtained from Kohn–Sham density functional theory (DFT) as implemented in the plane-wave pseudopotential code Quantum Espresso.^38^ We used norm-conserving Hartwigsen–Goedecker–Hutter pseudopotential^66^ with Perdew–Burke–Ernzerhof generalized gradient approximation (GGA-PBE) to the exchange-correlation functional.^67^ All calculations employed the DFT-relaxed crystal structure. The BZ is sampled with 12 × 12 × 4 mesh. The phonons are calculated with a **q**-grid of 5 × 5 × 2 in the BZ based on density functional perturbation theory (DFPT).^62^ Spin–orbital coupling is neglected in our calculations.

The electron–phonon coupling matrix elements are calculated within the EPW code,^68,69^ which uses Wannier90(63) as a module. The wave functions from DFT calculations are projected onto 22 maximally localized Wannier functions (MLWF)^63,64^ using d orbitals of Mo and *p* orbitals of S as initial projectors. The electron energies, phonon frequencies, and electron–phonon coupling matrix elements are interpolated on a 48 × 48 × 10 Monkhorst–Pack homogeneous mesh for both **k** and **q** points using MLWF. We used a smearing parameter of 3 meV for the calculation of the electron–phonon collision integral and 0.05 meV for the evaluation of the phonon–phonon collision integral. The third-order force constants—required for the evaluation of momentum- and mode-resolved phonon–phonon relaxation times—are obtained from finite differences using the third-order.py utility of the shengBTE code.^70^ Calculations are performed on a 3 × 3 × 2 supercell. Ab initio simulations of the ultrafast electron–phonon dynamics are based on TDBE, which we have implemented in the EPW code. The time derivative is computed using the second-order Runge–Kutta (Heun’s) method with a time step of 2 fs. The details of the collision integrals and the implementations are provided elsewhere.^51,52^

## Supporting Information Available

Comparison between simulations and experiments at all pump-probe delays (MP4)
Estimation of excited carrier density; nonequilibrium phonon dynamics with different initial carrier distributions; screened versus unscreened signals under different initial electronic temperatures; calculations of diffuse scattering maps; Bragg peak dynamics; screening of the EPC matrix elements; extraction of the momentum-resolved phonon dynamics in the experiments and theory; and anharmonic phonon scattering rate (PDF)

## Notes

A preprint version of this paper can be found under Y.P.; P.-N.H.; D.Z.; M.Z.; Y.W.W.; R.E.; F.C.; H.S. Momentum-Resolved Signatures of Carrier Screening Effects on Electron–Phonon Coupling in MoS_2_. 2025, 2503.03363. arXiv. arxiv.org/abs/2503.03363 (accessed March 7, 2025). The data presented here are available on a data repository.^71^
