# Supplementary material for: Momentum-Resolved Signatures of Carrier Screening Effects on Electron–Phonon Coupling in MoS2
Source: ACS Nano. 2025 Mar 15;19(11):11381–9. doi: 10.1021/acsnano.5c00744 (PMC11948618; doi:10.1021/acsnano.5c00744)
Supplement: Supplementary file 2 — nn5c00744_si_002.pdf [file nn5c00744_si_002.pdf]

Supporting Information: Momentum-Resolved Signatures of Carrier Screening Effects on Electron-Phonon Coupling in MoS_2

Yiming Pan,[†] Patrick-Nigel Hildebrandt,[‡] Daniela Zahn,[‡] Marios Zacharias,[¶] Yoav William Windsor,[‡,§] Ralph Ernstorfer,[‡,§] Fabio Caruso,[*,†] and Hélène Seiler[*,‡,‖]

†*Institut für Theoretische Physik und Astrophysik, Christian-Albrechts-Universität zu Kiel, 24118 Kiel, Germany*

‡*Fritz Haber Institute of the Max Planck Society, Faradayweg 4-6, 14195 Berlin, Germany*

¶*Univ Rennes, INSA Rennes, CNRS, Institut FOTON - UMR 6082, F-35000 Rennes, France*

§*Institut für Optik und Atomare Physik, Technische Universität Berlin, Strasse des 17. Juni 135, 10623, Berlin, Germany*

‖*Freie Universität Berlin, Arnimallee 14, 14195 Berlin, Germany*

E-mail: caruso@physik.uni-kiel.de; helene.seiler@fu-berlin.de

Contents

S1 Estimation of excited carrier density **3**

S2 Nonequilibrium phonon dynamics with different initial carrier distributions 4

S3 Screened versus unscreened signals under different initial electronic temperatures 8

S4 Calculations of diffuse scattering maps 10

S5 Bragg peak dynamics 13

S6 Screening of the EPC matrix elements 14

S7 Extraction of the momentum-resolved phonon dynamics in the experiments and theory 16

S8 Anharmonic phonon scattering rate 18

References 19

S1 Estimation of excited carrier density

The incident fluence on the MoS_2 flake is $I_{\text{inc}} = 44$ J/m^2. The complex refractive index of MoS_2 at 580 nm is 4.89 + 1.15i, as estimated from a previous work[S1]. We use transfer matrices to calculate the transmitted part, T, and the reflected part, R, of the incident fluence. This yields the absorbed fluence $I_{\text{abs}} = (1 - R - T) \cdot I_{\text{inc}}$. The carrier density per square centimeter is then:

$$n = 10^{-6} \cdot \frac{1}{E_{\text{ph}}} \cdot \frac{I_{\text{abs}}}{d} \cdot c \cdot \frac{1}{2} = (1.29 - 1.69) \cdot 10^{14} \quad \text{electrons/cm}^2,$$

where E_{ph} is the energy of one pump photon, d is the flake thickness, which we estimate to 33 ± 7 nm, $c = 13.98 \cdot 10^{-10}$ m is the unit cell length in the out-of-plane direction. The range of values for n arises from the error on the flake thickness.

In order to generate the initial carrier distribution for the TDBE simulations with the estimated carrier density, we simulated the hot carrier distributions with Fermi-Dirac functions $f_{n\mathbf{k}} = 1/(e^{\frac{\varepsilon_{n\mathbf{k}} - \mu}{k_B T_{\text{el}}}} + 1)$, where the electronic temperature is assumed to be 4000 K in the results shown in the main text. The chemical potentials μ for electrons (conduction bands) and holes (valence bands) are determined by solving the nonlinear equation $n = \frac{2}{S N_{\mathbf{k}}} \sum_{n\mathbf{k}} f_{n\mathbf{k}}$, where n is the carrier density with a value matching the experimental excitation density, the factor 2 accounts for the spin degeneracy, S is the in-plane area of the unit cell of bulk MoS_2 and $N_{\mathbf{k}}$ is the number of $\mathbf{k}$ points used to sample the BZ.

S2 Nonequilibrium phonon dynamics with different initial carrier distributions

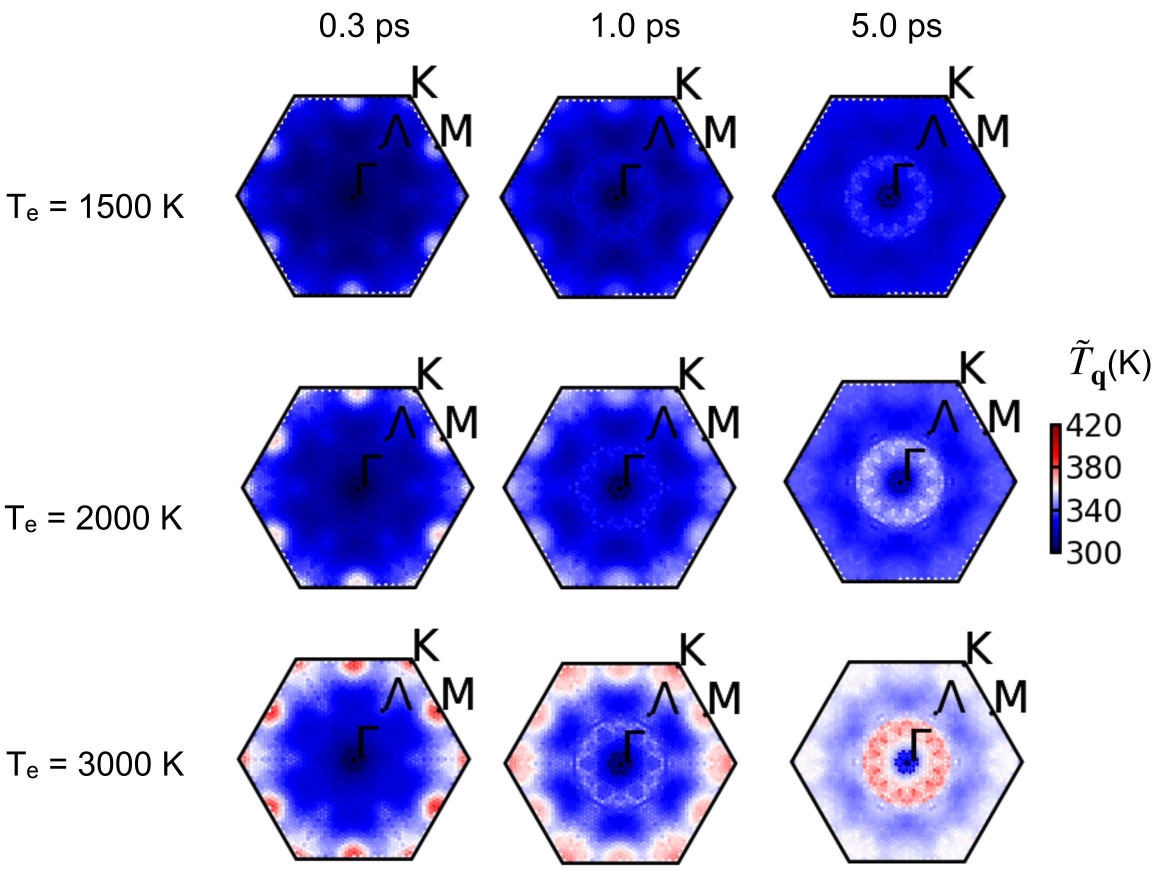

Figure S1: Momentum-resolved phonon temperature $\tilde{T}_{\mathbf{q}}$ averaged over phonon branches at 0.3 ps, 1 ps and 5 ps for initial carrier temperatures set at 1500 K, 2000 K and 3000 K, for comparison with 4000 K presented in the main text.

In the following we present simulations with different initial electronic temperatures. Carriers are distributed in a broader (narrower) range of energy with a higher (lower) electronic temperature, as demonstrated in Fig. S4. However, the phonon emission pathways remain essentially unchanged, which leads to qualitatively similar results, as reported in Fig. S1, Fig. S2 and Fig. S3 for $T_{\mathrm{el}} = 1500,\ 2000,\ 3000$ K.

Since our maximum simulation time is 5 ps, where the lattice is still in a non-thermal

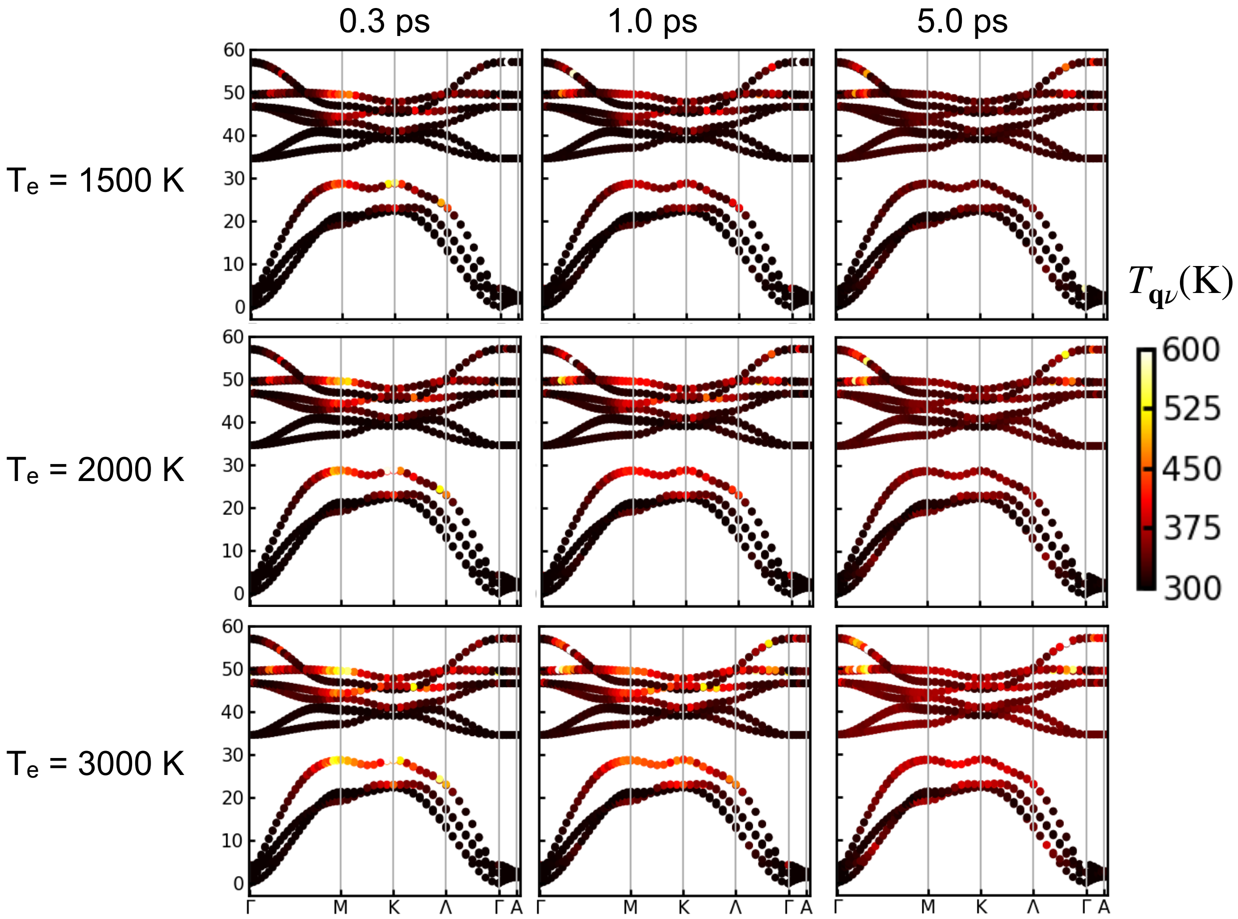

Figure S2: Momentum- and branch- resolved phonon temperature $T_{\mathbf{q}\nu}$ at 0.3 ps, 1 ps and 5 ps for initial carrier temperatures set at 1500 K, 2000 K and 3000 K, for comparison with 4000 K presented in the main text.

state, we cannot directly extract the temperature rise of the lattice from the simulations. Nevertheless, at a pump-probe delay of 5 ps, the energy transfer rate between electrons and the lattice is already low, which can be seen from the time-dependent lattice energy, evaluated with harmonic approximation:

$$E_{\text{latt}}(t) = N_p^{-1} \sum_{\mathbf{q}\nu} \hbar\omega_{\mathbf{q}\nu} n_{\mathbf{q}\nu}(t) \tag{S1}$$

The energy of the lattice tends towards its equilibrium energy even if the phonon subsystem is still not thermalized. On a 5-50 ps time scale, the energy redistributed within the phonon subsystem until thermalization is reached. As a result, we can estimate the final

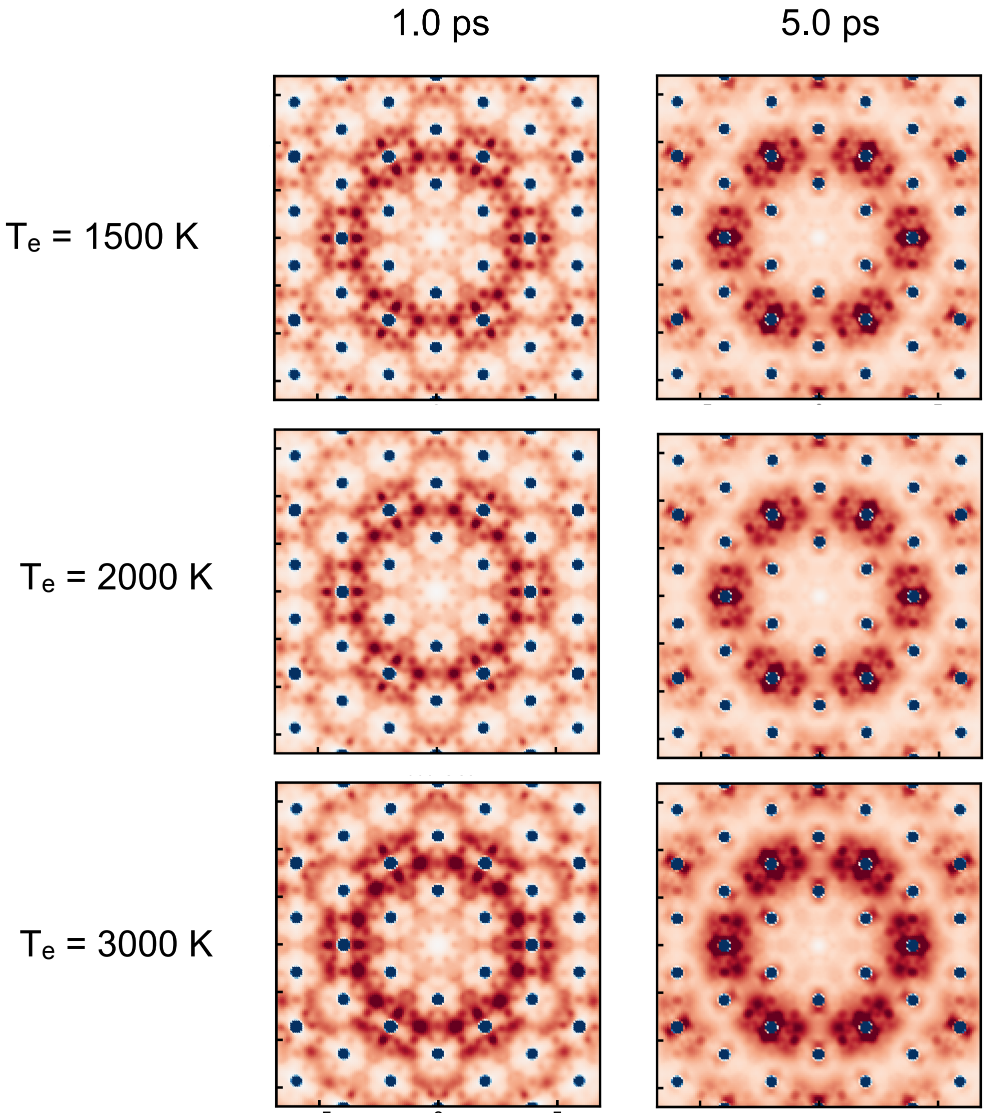

Figure S3: Calculated diffuse scattering signals $\Delta I(\mathbf{Q}, t) = I(\mathbf{Q}, t) - I(\mathbf{Q}, t \leq t_0)$ at pump-probe delays of 1 ps and 5 ps using different electronic temperature as initial conditions of carrier distribution.

temperature of the lattice by comparing the time-dependent lattice energy with the lattice energy at a certain temperature:

$$E_{\rm latt}(T) = N_p^{-1} \sum_{\mathbf{q}\nu} \frac{\hbar\omega_{\mathbf{q}\nu}}{e^{\hbar\omega_{\mathbf{q}\nu}/k_b T} - 1} \tag{S2}$$

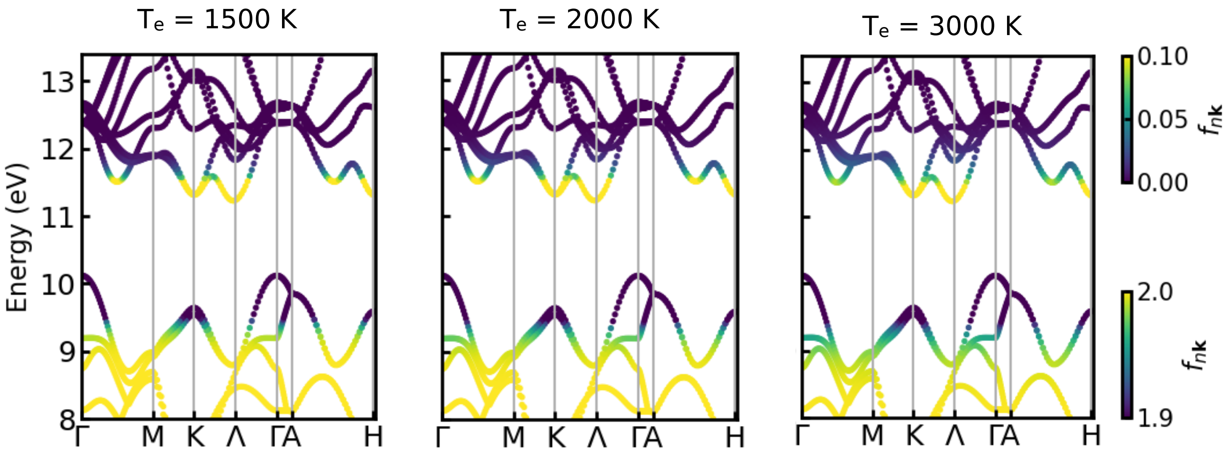

Figure S4: Distribution of excited electrons and holes in the conduction and valence band of MoS_2, marked as a color coding superimposed to the electron bands, for different initial effective electronic temperatures.

By equating these two expressions, we can estimate the rise in lattice temperature. The time-dependent lattice energy is reported in Fig. S5. The initial electronic temperature of 4000 K is thus chosen such that the ensuing rise in lattice temperature matches the experimental estimate of 70 K.

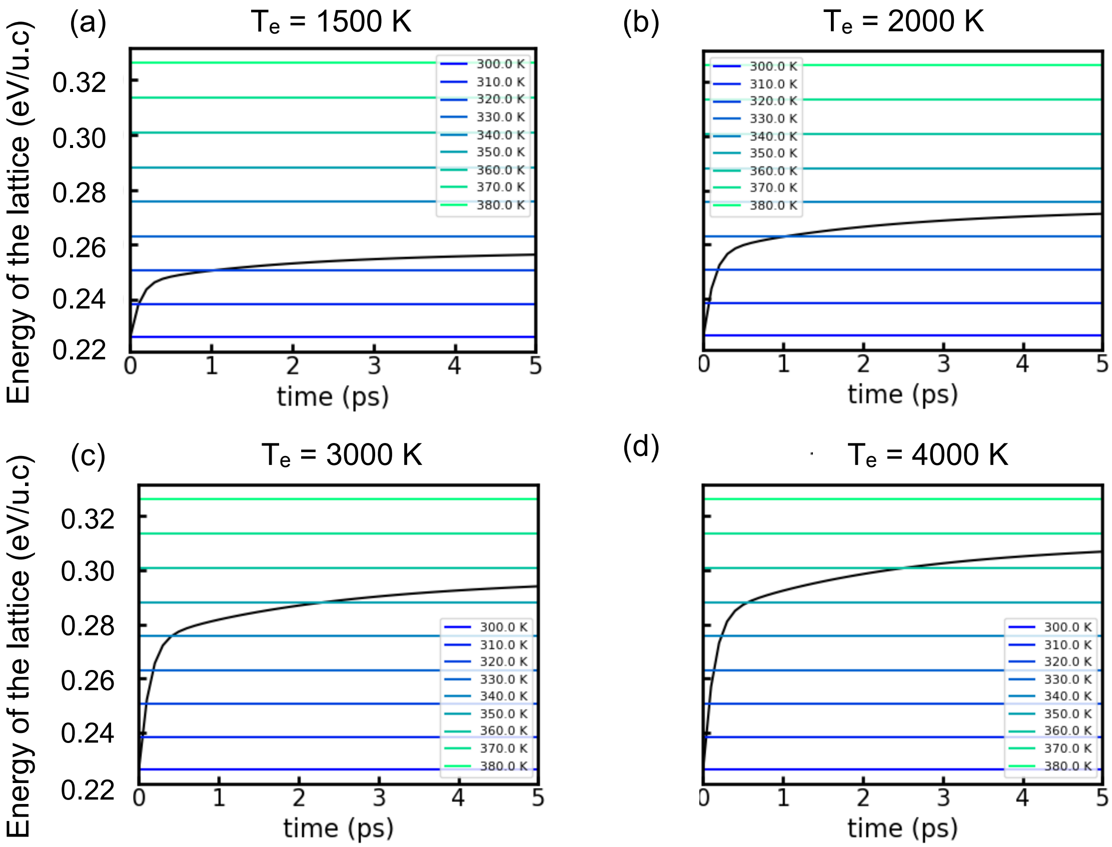

Figure S5: Energy of the lattice evaluated from Eq. (S1). The horizontal lines mark the energy of a thermal lattice from 300 K to 380 K.

S3 Screened versus unscreened signals under different initial electronic temperatures

In Fig. S6(a)-(b), we compare zoom-ins of the experimental diffuse scattering maps around Bragg peak (110) at 1 ps and 2 ps to simulations with screened and unscreened electron-phonon coupling under different initial conditions for carrier distribution (T_e). We find that the effect of screening is visible regardless of the initial condition. The diffuse scattering maps simulated with screened electron-phonon coupling are in better agreement with the experimental data close to Γ (Bragg peaks) compared to the unscreened results. The simulated intensity is reported in Fig. S6(c)-(f) for crystal momenta along a path crossing the Bragg

peak (marked by the green dashed line in Fig. 2 of the main text), as well as in Fig. S7 compared to experimental results. The maximum of the intensity with screened electron-phonon coupling appears further away from the Γ point, as opposed to the results with unscreened electron-phonon coupling, which find their maximum at a smaller wavevector, indicating a higher phonon population for low |**q**| phonons than what experiments suggest.

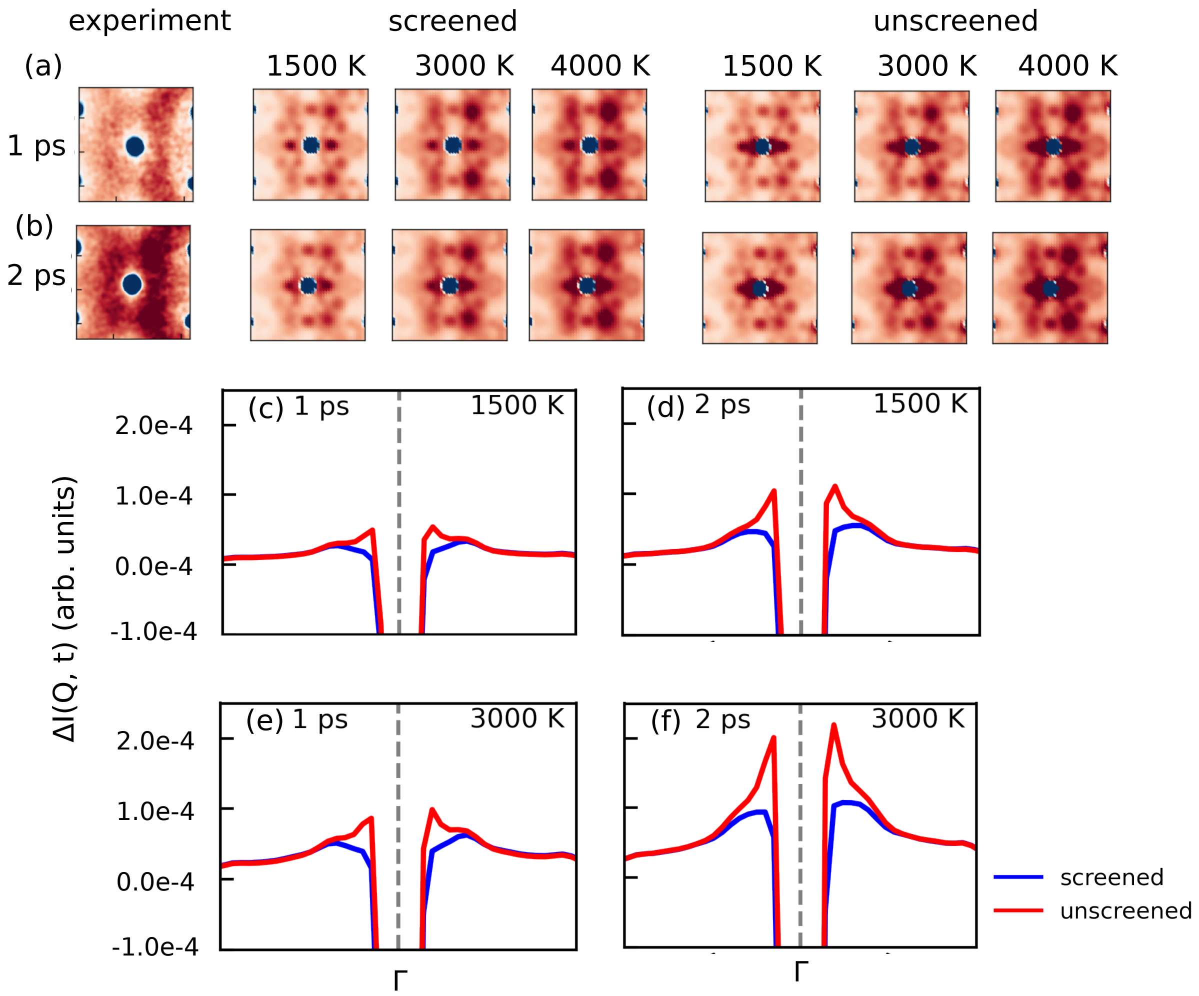

Figure S6: Zoom-in on the Brillouin zone around Bragg peak (110) of the experimental results and simulations with screened and unscreened electron-phonon coupling with different initial electronic distributions at 1 ps (a) and 2 (ps) (b). The differential intensity obtained from the two types of simulations (screened and unscreened) are plotted together along the path crossing Bragg peak (110), shown by the green dashed line in Fig. 2 of the main text, for T_e = 1500 K (c)-(d) and T_e = 3000 K (e)-(f).

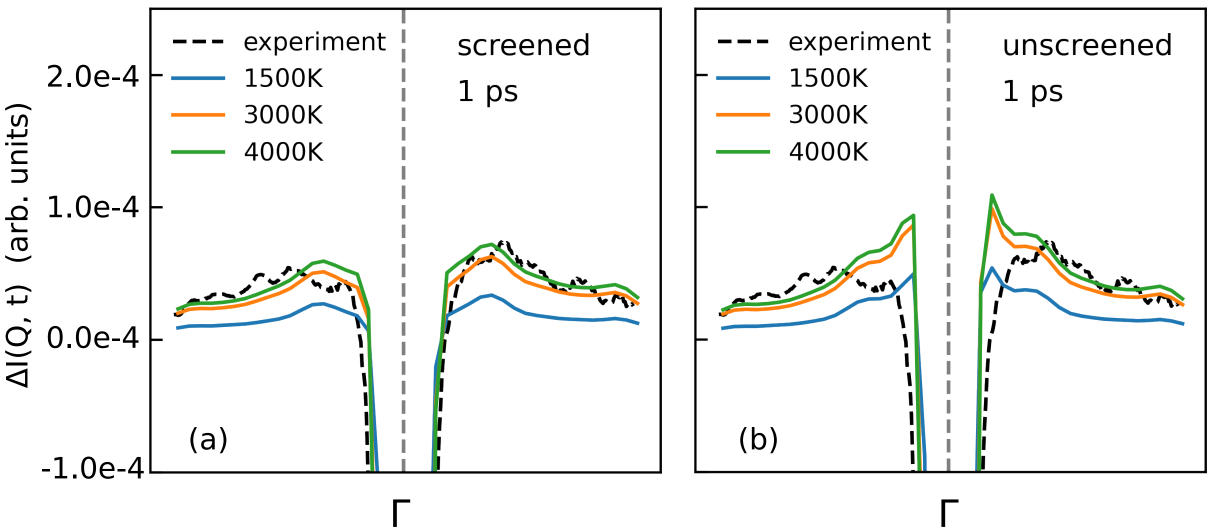

Figure S7: Differential diffuse scattering intensity at 1 ps with screened (a) and unscreened (b) electron-phonon coupling with initial electronic temperatures of 1500 K, 3000 K and 4000 K. The experimental signal is plotted as a black dotted line for comparison with simulations.

S4 Calculations of diffuse scattering maps

We calculate the diffuse scattering intensity for nonthermal phonons based on quantum-mechanical scattering theory, in order to account for all phonon contributions to inelastic scattering processes. The scattering intensity is calculated using the code `disca.x`, which is available in the subpackage `ZG` of the `EPW` code.[S2,S3] The all-phonon structure factor includes the contribution of multiphonon effects $I_{\mathrm{all}}(\mathbf{Q}, t)$ and is calculated according to the following expression:

$$I_{\mathrm{all}}(\mathbf{Q}, t) = N_p \sum_{p} \sum_{\kappa,\kappa'} f_\kappa(\mathbf{Q}) f^{\star}_{\kappa'}(\mathbf{Q}) e^{i\mathbf{Q}\cdot[\mathbf{R}_p + \boldsymbol{\tau}_\kappa - \boldsymbol{\tau}_{\kappa'}]} e^{-W_\kappa(\mathbf{Q},t)} e^{-W_{\kappa'}(\mathbf{Q},t)} e^{P_{p,\kappa\kappa'}(\mathbf{Q},t)} \tag{S3}$$

Where N_p is the number of $\mathbf{q}$ points used to sample the first Brillouin Zone, $f_\kappa(\mathbf{Q})$ is the atomic form factor of atom κ, and can be evaluated using the methods and parameters from Ref. S4. $\boldsymbol{\tau}_\kappa$ denotes the position of atoms in the unit cell and $\mathbf{R}_p$ is the position of unit-cell in a Born-von Kármán supercell. $W_\kappa(\mathbf{Q}, t)$ is the Debye-Waller factor of atom κ.

The phononic factor $e^{P_{p,\kappa\kappa'}(\mathbf{Q},t)}$ includes contributions of phonon scattering of all orders. The time-dependence of the Debye-Waller factor and the phononic factor are encoded in the mean-square displacements. These factors can be described by the following expressions:

$$P_{p,\kappa\kappa'}(\mathbf{Q},t) = \frac{M_0 N_p^{-1}}{\sqrt{M_\kappa M_{\kappa'}}} \sum_{\mathbf{q}\nu} \langle u_{\mathbf{q}\nu}^2 \rangle \mathrm{Re}\big[\mathbf{Q}\cdot\mathbf{e}_{\mathbf{q}\nu}^{\kappa}\mathbf{Q}\cdot\mathbf{e}_{\mathbf{q}\nu}^{\star\kappa'} e^{i\mathbf{q}\cdot\mathbf{R}_p}\big] \tag{S4}$$

$$W_\kappa(\mathbf{Q},t) = \frac{M_0}{2N_p M_\kappa} \sum_{\mathbf{q}\nu} \langle u_{\mathbf{q}\nu}^2 \rangle |\mathbf{Q}\cdot\mathbf{e}_{\mathbf{q}\nu}^{\kappa}|^2 \tag{S5}$$

where M_0 and M_κ are reference and atomic masses. The phonons are described by the eigenvectors $\mathbf{e}_{\mathbf{q}\nu}$ and the frequencies $\hbar\omega_{\mathbf{q}\nu}$. The mean-square displacement of mode $\mathbf{q}\nu$ is defined as $\langle u_{\mathbf{q}\nu}^2(t)\rangle = \frac{\hbar}{M_0\omega_{\mathbf{q}\nu}}\big[n_{\mathbf{q}\nu}(t) + \frac{1}{2}\big]$. The phonon wave vector $\mathbf{q}$ is summed over the Brillouin zone in Eq. (S4) and Eq. (S5).

Expanding the exponential of the phononic factor $e^{P_{p,\kappa\kappa'}}$ enables us to decompose the diffraction intensity as $I(\mathbf{Q},t) = I_0(\mathbf{Q},t) + I_1(\mathbf{Q},t) + \cdots$, where the zeroth order $I_0(\mathbf{Q},t)$ describes the Bragg scattering diminished by thermal effects and is nonzero only at the Bragg peak positions, *i.e.* $\mathbf{Q} = \mathbf{G}$. The first-order term $I_1(\mathbf{Q},t)$ accounts for a large proportion of diffuse scattering intensity and can be evaluated by

$$I_1(\mathbf{Q},t) \propto \sum_{\nu} \frac{n_{\mathbf{q}\nu} + 1/2}{\omega_{\mathbf{q}\nu}} \Big| \sum_{\kappa} e^{-W_\kappa(\mathbf{Q}) - i\mathbf{Q}\cdot\boldsymbol{\tau}_\kappa} \frac{f_\kappa(\mathbf{Q})}{\sqrt{M_\kappa}} \big(\mathbf{Q}\cdot\mathbf{e}_{\mathbf{q}\nu}^{\kappa}\big)\Big|^2 \tag{S6}$$

where the scalar product can be used as a selection rule to distinguish the contribution from different phonon branches. In addition, the scattering vector $\mathbf{Q}$ and the phonon wave vector $\mathbf{q}$ only differ by a reciprocal lattice vector, *i.e.* $\mathbf{Q} = \mathbf{q} + \mathbf{G}$.

Finally, we make use of the selection rule $(\mathbf{Q}\cdot\mathbf{e}_{\mathbf{q}\nu}^{\kappa})$ in the expression for the diffuse scattering intensity, to resolve some fine features of the diffuse scattering maps in Fig. 2 of the main text. In this scalar product, $\mathbf{e}_{\mathbf{q}\nu}^{\kappa}$ is the phonon eigenvector projected on atom κ. The scattering vector $\mathbf{Q}$ and phonon wave vector $\mathbf{q}$ differ by a reciprocal lattice vector.

The scalar product acts as a selection rule to distinguish contributions from different phonon branches. For instance, the population of the strongly coupled LA(M) phonon contributes to the intensity at the midpoint of Bragg peaks (010) and (020), since the scattering vector $\mathbf{Q}$ is parallel to the eigenvector of the LA(M) phonon. This point displays a visible red spot at 1 ps, which slowly decreases in intensity at 5 ps until thermal equilibrium, as demonstrated by the diffuse scattering maps in Fig. 2 of the main text. This observation is consistent with the temporal evolution of the LA(M) population revealed in Fig. 5(f) and (g) of the main text. Similarly, the TA phonon contributes to the scattering intensity for $\mathbf{Q}$ vectors (close to) orthogonal to the wave vector $\mathbf{q}$. The intensity for the M point at the midpoint between Bragg peaks (020) and (110) therefore mainly arises from the TA(M) phonon, which grows slowly until 5 ps as a result of weak electron-phonon coupling. The main difference between the diffuse scattering signals at 5 ps and 50 ps is the formation of the starlike shape joining the Bragg peaks in the diffuse scattering in thermal equilibrium, which is attributed to the temperature anisotropy of acoustic phonons along the Γ-M line at the second nonthermal stage.

S5 Bragg peak dynamics

Overall 48 Bragg peaks were analyzed, grouped in 8 families. In Fig. S8 we show the response of the different Bragg peak families. Fig. S9 displays the average elastic scattering response as obtained by averaging over all peaks. The trace can be fitted with a bi-exponential function convolved with a Gaussian of 150 fs duration, yielding a fast time constant $\tau_1 = 470 \pm 20$ fs associated with amplitude $A_1 = -0.043 \pm 0.001$, and a slower time constant $\tau_2 = 2.8 \pm 0.2$ ps with a smaller amplitude $A_2 = -0.011 \pm 0.001$. The two components are shown as grey dashed lines in Fig. S9. The first time constant τ_1 is consistent with previous optical spectroscopy results performed at similar excitation densities on multilayer MoS_2 , where the time constant was assigned to the electron-phonon scattering time[S5]. The second time constant τ_2 is on the same order of magnitude as the phonon-phonon equilibration time reported in previous diffraction studies on (supported) monolayer MoS_2[S6–S9]. While the presence of two-step behaviour already suggests the presence of nonthermal phonon populations, no direct information can be obtained on carrier screening and its impact on the lattice dynamics from the Bragg peak dynamics.

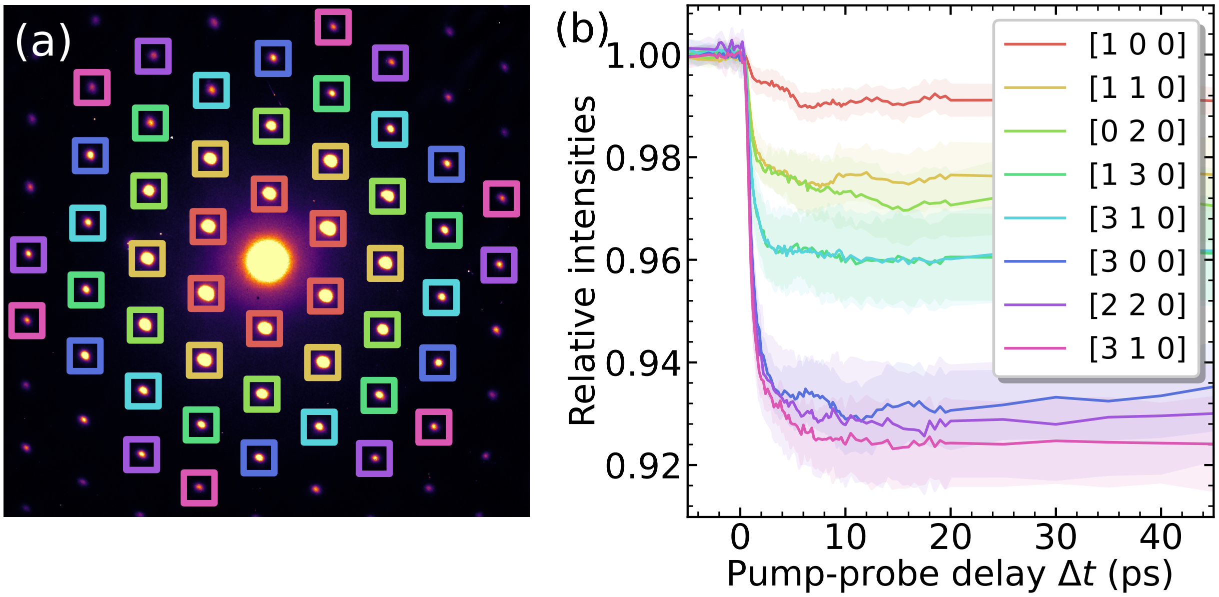

Figure S8: (a) Diffraction pattern of MoS_2 with colored rectangles indicating the peaks which were analyzed. A given color corresponds to a given peak family. (b) Bragg peaks dynamics as a function of pump-probe delay for the different peak families. Each trace shows the average over peaks belonging to the same family. The color code matches the one of panel (a).

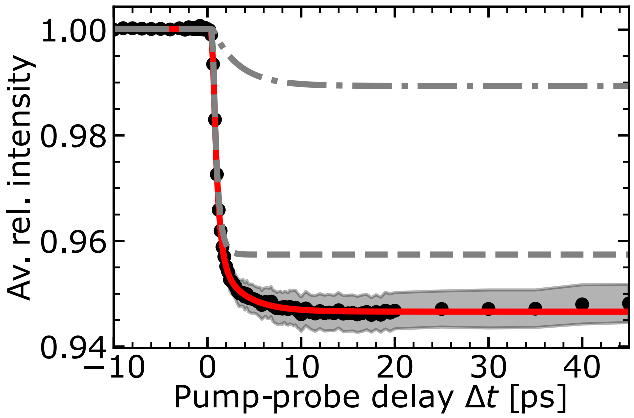

Figure S9: Elastic scattering response obtained by averaging over the 48 Bragg peaks shown in Fig. S8. Experimental data (black circles), bi-exponential fit (red solid line), and individual fit components (dashed grey lines).

S6 Screening of the EPC matrix elements

The EPCs are screened with static dielectric functions via the following expression:

$$\tilde{g}_{mn\nu}(\mathbf{k},\mathbf{q}) = g_{mn\nu}(\mathbf{k},\mathbf{q})\frac{\epsilon_{\text{undop}}(\mathbf{q})}{\epsilon_{\text{dop}}(\mathbf{q})} \tag{S7}$$

Where the difference between the doped (ϵ_{undop}) and undoped dielectric function (ϵ_{undop}) accounts for the contribution of photoexcited carriers. In the independent particle approximation, it can be expressed as:

$$\epsilon_{\text{dop}}(\mathbf{q}) = \epsilon_{\text{undop}}(\mathbf{q}) - \frac{2}{N_{\mathbf{k}}} \frac{4\pi e^2}{|\mathbf{q}|^2} \sum_{mm'\mathbf{k}} \frac{\delta f_{m\mathbf{k}} - \delta f_{m'\mathbf{k}+\mathbf{q}}}{\varepsilon_{m\mathbf{k}} - \varepsilon_{m'\mathbf{k}+\mathbf{q}}} |\langle u_{m\mathbf{k}}|u_{m'\mathbf{k}+\mathbf{q}}\rangle|^2 \tag{S8}$$

As a result, the screening function defined as $\epsilon_{\text{M}}(\mathbf{q}) \equiv \frac{\epsilon_{\text{dop}}(\mathbf{q})}{\epsilon_{\text{undop}}(\mathbf{q})}$ coincides with Eq. (1). We use the electronic energies and eigenvectors obtained from Wannier interpolations to evaluate the dielectric functions.

In order to account for the dynamical effects in the screening, one needs to account for the phonon energy in the denominator of Eq. (S8) by making the replacement $\varepsilon_{m\mathbf{k}} - \varepsilon_{m'\mathbf{k}+\mathbf{q}} \rightarrow \varepsilon_{m\mathbf{k}} - \varepsilon_{m'\mathbf{k}+\mathbf{q}} - \hbar\omega_{\mathbf{q}\nu}$. However, our calculations of the screening function show very little difference when the phonon energy is taken into account, as illustrated by the inverse of the screening function, *i.e.* $\epsilon_{\text{M}}^{-1}(\mathbf{q})$ in Fig. S10.

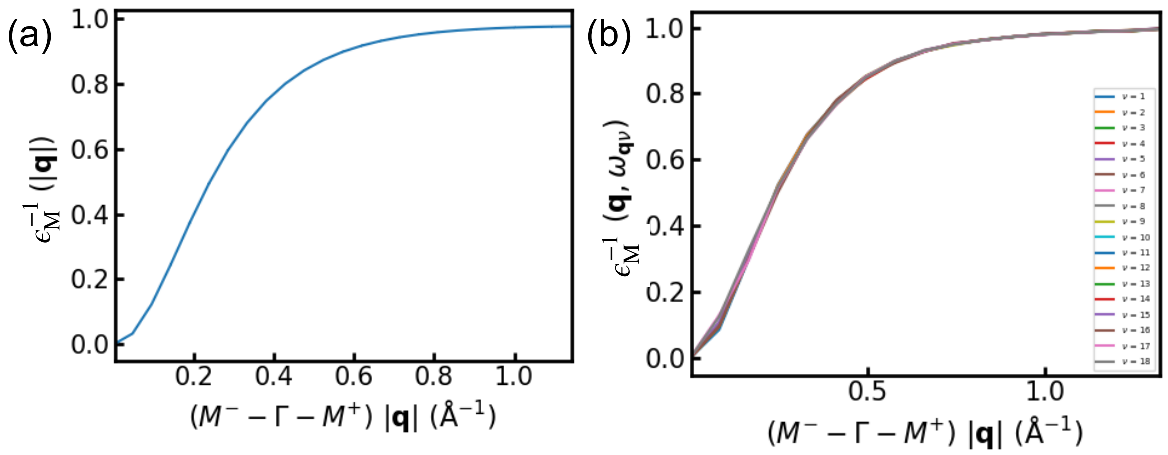

Figure S10: Different dielectric functions evaluated on the $\Gamma - M$ high-symmetry path of the BZ. (a) Static dielectric function. (b) Dynamic dielectric function at the frequency of phonons.

S7 Extraction of the momentum-resolved phonon dynamics in the experiments and theory

Each of the Bragg peaks (blue dots) in Fig. 2 of the main text and Fig. S11(a) can be identified with the center of the first Brillouin Zone (BZ). This is justified from the ordinary scattering theory for a static lattice, where the constructive interference condition leading to the emergence of the Bragg peaks is only obeyed for crystal momenta coinciding with a reciprocal lattice vector. The hexagonal Brillouin zone can be defined through the construction of the Voronoi cell around each of the Bragg peaks, enabling the identification of the high-symmetry points of the hexagonal unit cell. The entire diffraction pattern can be "tiled" by connected BZs, see for example the 3 BZs drawn on Fig. S11. Each Bragg peak is located at the Γ point of the BZ, and other high-symmetry points can also be labeled on the diffraction pattern, such as K and M, see Fig. 2(a) of the main text. The momentum resolution of FEDS allows for a clear distinction between signals arising from long-wavelength and short-wavelength phonons. Long-wavelength phonons, characterized by small wavevectors, are located near the Γ point, whereas short-wavelength phonons, with larger wavevectors, are found in the outer regions of the BZ.

As described in detail in Refs. S2 and S3, the inelastic scattering signals shown in Fig. 2 of the main text and Fig. S11 look different from BZ to BZ due to modulations by the structure factor. However, the phonon population dynamics, of interest here, are redundant in all BZs. Therefore, we implement a data analysis procedure such that the signals of all observed BZs can be averaged. From the raw diffraction patterns as shown in Fig. S11(a), we first correct for experimental distortions in the diffraction patterns arising from our magnetic lens. We then employ a Monkhorst-Pack sampling of the diffraction pattern, which ensures the same sampling point distributions in every BZ. Fig. S11(b) shows an example sample grid for the 3 BZs of panel (a), matching colors. Once this sampling is completed, the data of all different BZs can be averaged into a single BZ, seen in Fig. S11(c). The signals at various

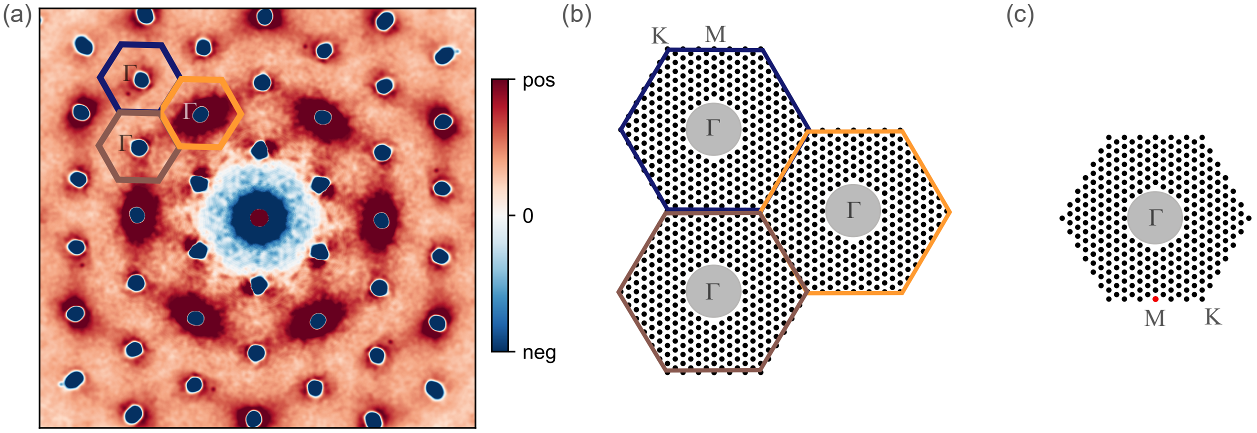

Figure S11: Key steps for the extraction of momentum-resolved phonon dynamics in the experiments. (a) Differential diffuse scattering signal for 50 ps. The diffraction pattern can be "tiled" with BZs, as illustrated with the 3 exemplary BZs. (b) The diffraction pattern is sampled using a Monkhorst-Pack grid, ensuring each BZ in the pattern is sampled the same way as other BZs. (c) Signals from all the different BZs can be averaged into a single BZ. Phonon dynamics at a particular reduced wavevector **q** - for example at the M point shown in red - can then be extracted by summing detector counts around that point.

points of the BZ, for example at the M point shown in red, can be obtained by using a circular integration window and summing the detector counts contained in this window. Doing this for every pump-probe delay yields time-resolved traces. This data analysis method makes the most of the available data by using all available BZs, reduces the arbitrariness associated with picking a particular Bragg peak, and significantly increases the signal-to-noise compared to signals obtained around a single Bragg peak. The relative intensity at reduced wavevector $\mathbf{q}$ is then obtained by $I_r(\mathbf{q},t) = \frac{I(\mathbf{q},t)-I(\mathbf{q},t<t_0)}{I(\mathbf{q},t=50ps)-I(\mathbf{q},t<t_0)}$.

For the simulated results, the relative intensity is averaged over 48 Brillouin zones, and calculated according to the expression $I_r(\mathbf{q},t) = \frac{I(\mathbf{q},t)-I(\mathbf{q},T=300\text{ K})}{I(\mathbf{q},T=370\text{ K})-I(\mathbf{q},T=300\text{ K})}$, where we estimate the lattice temperature rises from 300 K to 370 K. The temperature rise of the lattice is evaluated by calculating the energy of the lattice in each time step of the simulation, which tends towards the energy of the lattice at 370 K.

S8 Anharmonic phonon scattering rate

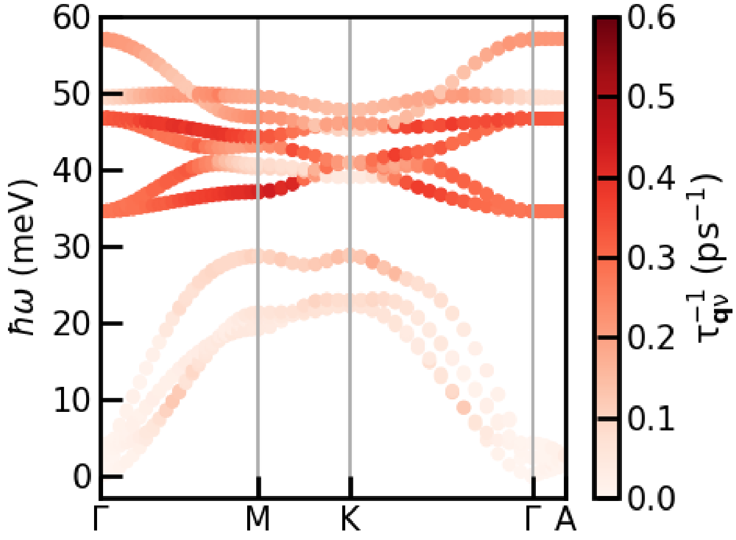

Figure S12: Phonon scattering rate due to phonon-phonon interactions evaluated at 300 K.

The thermalization of the nonthermal lattice relies on phonon-phonon interactions, reflected by the scattering rate (inverse lifetime) of phonon mode $\mathbf{q}\nu$. Accounting for the lowest order, the anharmonic Hamiltonian is:

$$\hat{H}_{\text{anh}} = \frac{1}{3!} \sum_{\mathbf{qq'q''},\nu\nu'\nu''} \Psi^{\nu\nu'\nu''}_{\mathbf{qq'q''}} (\hat{a}_{\mathbf{q}\nu} + \hat{a}^{\dagger}_{-\mathbf{q}\nu})(\hat{a}_{\mathbf{q'}\nu} + \hat{a}^{\dagger}_{-\mathbf{q'}\nu})(\hat{a}_{\mathbf{q''}\nu} + \hat{a}^{\dagger}_{-\mathbf{q''}\nu}) \tag{S9}$$

, and the scattering rate can be calculated according to[S10]:

$$\tau^{-1}_{\mathbf{q}\nu} = \frac{\pi\hbar}{N_{\mathbf{q}}} \sum_{\mathbf{q'}\nu'\nu''} |\Psi^{\nu\nu'\nu''}_{\mathbf{qq'q''}}|^2 \big[(1 + n_{\mathbf{q'}\nu'} + n_{\mathbf{q''}\nu''})\delta(\omega_{\mathbf{q}\nu} - \omega_{\mathbf{q'}\nu'} - \omega_{\mathbf{q''}\nu''}) \\ + 2(n_{\mathbf{q''}\nu''} - n_{\mathbf{q'}\nu'})\delta(\omega_{\mathbf{q'}\nu'} - \omega_{\mathbf{q''}\nu''} - \omega_{\mathbf{q}\nu})\big] \tag{S10}$$

where the phonon-phonon coupling matrix elements $\Psi^{\nu\nu'\nu''}_{\mathbf{qq'q''}}$ are interpolated from third-order force constants. For simplicity, we evaluate the above expression for a thermally

distributed phonon at 300 K in Fig. S12 , in order to provide a qualitative understanding of the thermalization rate. We note that our TDBE simulation is based on the evaluation of phonon-phonon collision integrals without using scattering rate, as described in a previous work by some of us.[S11]

References

[S1] Ermolaev, G. A.; Stebunov, Y. V.; Vyshnevyy, A. A.; Tatarkin, D. E.; Yakubovsky, D. I.; Novikov, S. M.; Baranov, D. G.; Shegai, T.; Nikitin, A. Y.; Arsenin, A. V.; Volkov, V. S. Broadband optical properties of monolayer and bulk MoS2. *npj 2D Mater. Appl.* **2020**, *4*, 21.

[S2] Zacharias, M.; Seiler, H.; Caruso, F.; Zahn, D.; Giustino, F.; Kelires, P. C.; Ernstorfer, R. Multiphonon diffuse scattering in solids from first principles: Application to layered crystals and two-dimensional materials. *Phys. Rev. B* **2021**, *104*, 205109.

[S3] Zacharias, M.; Seiler, H.; Caruso, F.; Zahn, D.; Giustino, F.; Kelires, P. C.; Ernstorfer, R. Efficient First-Principles Methodology for the Calculation of the All-Phonon Inelastic Scattering in Solids. *Phys. Rev. Lett.* **2021**, *127*, 207401.

[S4] Peng, L.-M. Electron atomic scattering factors and scattering potentials of crystals. *Micron* **1999**, *30*, 625–648.

[S5] Nie, Z.; Long, R.; Sun, L.; Huang, C. C.; Zhang, J.; Xiong, Q.; Hewak, D. W.; Shen, Z.; Prezhdo, O. V.; Loh, Z. H. Ultrafast carrier thermalization and cooling dynamics in few-layer MoS2. *ACS Nano* **2014**, *8*, 10931–10940.

[S6] He, X.; Chebl, M.; Yang, D. S. Cross-Examination of Ultrafast Structural, Interfacial, and Carrier Dynamics of Supported Monolayer MoS2. *Nano Lett.* **2020**, *20*, 2026–2033.

[S7] Mannebach, E. M. et al. Dynamic Structural Response and Deformations of Monolayer

MoS2 Visualized by Femtosecond Electron Diffraction. *Nano Lett.* **2015**, *15*, 6889–6895.

[S8] Britt, T. L.; Li, Q.; René de Cotret, L. P.; Olsen, N.; Otto, M.; Hassan, S. A.; Zacharias, M.; Caruso, F.; Zhu, X.; Siwick, B. J. Direct View of Phonon Dynamics in Atomically Thin MoS_2. *Nano Lett.* **2022**, *22*, 4718–4724.

[S9] Caruso, F. Nonequilibrium Lattice Dynamics in Monolayer MoS2. *J. Phys. Chem. Lett.* **2021**, *12*, 1734–1740.

[S10] Li, W.; Carrete, J.; A. Katcho, N.; Mingo, N. ShengBTE: A solver of the Boltzmann transport equation for phonons. *Comput. Phys. Commun.* **2014**, *185*, 1747–1758.

[S11] Pan, Y.; Caruso, F. Vibrational Dichroism of Chiral Valley Phonons. *Nano Lett.* **2023**, *23*, 7463–7469.
